# Psychosocial characteristics as potential predictors of suicide in adults: an overview of the evidence with new results from prospective cohort studies

**DOI:** 10.1038/s41398-017-0072-8

**Published:** 2018-01-22

**Authors:** G. David Batty, Mika Kivimäki, Steven Bell, Catharine R. Gale, Martin Shipley, Elise Whitley, David Gunnell

**Affiliations:** 1 Department of Epidemiology and Public Health, University College London, London, UK; 2 Department of Public Health and Primary Care, University of Cambridge, Cambridge, UK; 3 Centre for Cognitive Ageing & Cognitive Epidemiology, University of Edinburgh, Edinburgh, UK; 4 MRC Lifecourse Epidemiology Unit, University of Southampton, Southampton, UK; 5 MRC/CSO Social and Public Health Sciences Unit, University of Glasgow, Glasgow, UK; 6 School of Social and Community Medicine, University of Bristol, Bristol, UK; 7 National Institute of Health Research Biomedical Research Centre at the University Hospitals Bristol NHS Foundation Trust, Bristol, UK

## Abstract

In this narrative overview of the evidence linking psychosocial factors with future suicide risk, we collected results from published reports of prospective studies with verified suicide events (mortality or, less commonly, hospitalisation) alongside analyses of new data. There is abundant evidence indicating that low socioeconomic position, irrespective of the economic status of the country in question, is associated with an increased risk of suicide, including the suggestion that the recent global economic recession has been responsible for an increase in suicide deaths and, by proxy, attempts. Social isolation, low scores on tests of intelligence, serious mental illness (both particularly strongly), chronic psychological distress, and lower physical stature (a marker of childhood exposures) were also consistently related to elevated suicide rates. Although there is some circumstantial evidence for psychosocial stress, personality disposition, and early-life characteristics such as bullying being risk indices for suicide, the general paucity of studies means it is not currently possible to draw clear conclusions about their role. Most suicide intervention strategies have traditionally not explored the modification of psychosocial factors, partly because evidence linking psychosocial factors with suicide risk is, as shown herein, largely in its infancy, or, where is does exist, for instance for intelligence and personality disposition, the characteristics in question do not appear to be easily malleable.

## Global burden of suicide

While the rate of suicide is declining worldwide, there are around 800,000 deaths from suicide per annum resulting in this behaviour being ranked the 15th leading cause of death^1^. Scrutinisation of the frequency of the intentional ending of one’s own life by age is striking: suicide is the second most common cause of death in 15–29 years old, and fifth in people aged 30–49 years^1^. With estimates suggesting that an additional 30–40 attempts on life are made across all age groups (100 in younger people) for every completed suicide in well-resourced countries^2^, the public health implications of such events are clearly burdensome, equating to more than 20 million attempted suicides per annum worldwide.

**Fig. 1:**
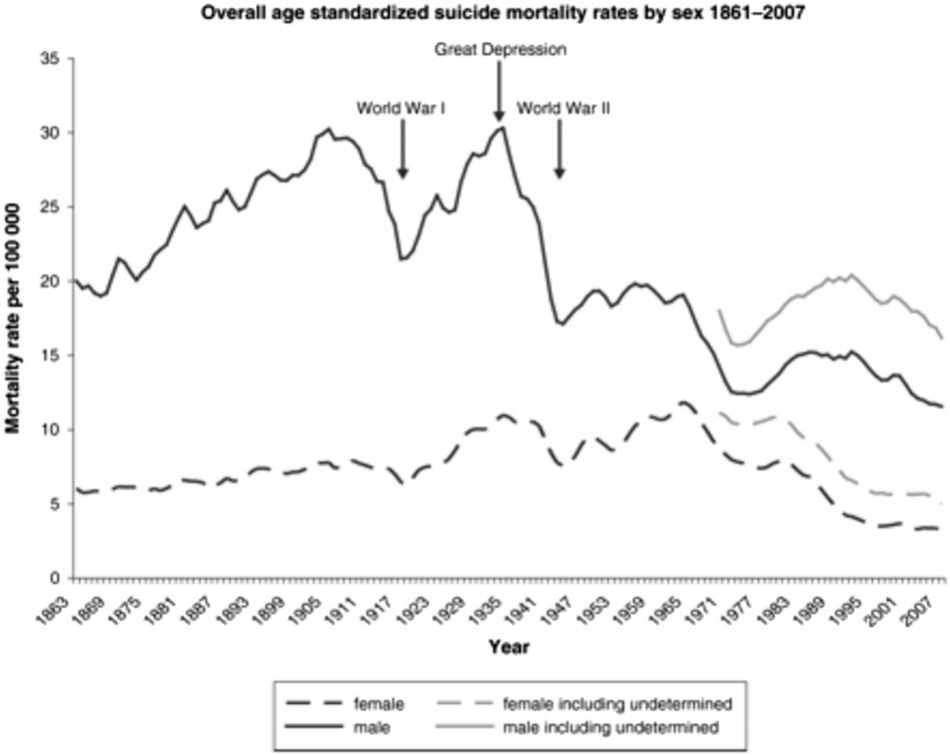
Annual suicide rates (age-standardised) for ages ≥15 years in England and Wales (3-year moving averages) 1861–2007 *Source*: Suicide in England and Wales 1861–2007: a time-trends analysis^3^. Reproduced with permission following payment to OUP

Division of suicide rates by epoch, gender, and region is also informative. Time series analyses of suicide deaths in the last 150 years in England and Wales, for instance, confirm that, whereas rates in men and women are at an all-time low, there are striking historical variations (Fig. 1)^3^. In men, for instance, the highest rates of completed suicide occurred in 1905 and 1934 (circa Great Depression), with marked declines during the two world wars (1914–1918, 1939–1945). Subsequently, against a general downward trend, there were some modest inflections in the 1950 and 1980s^3^.

**Fig. 2:**
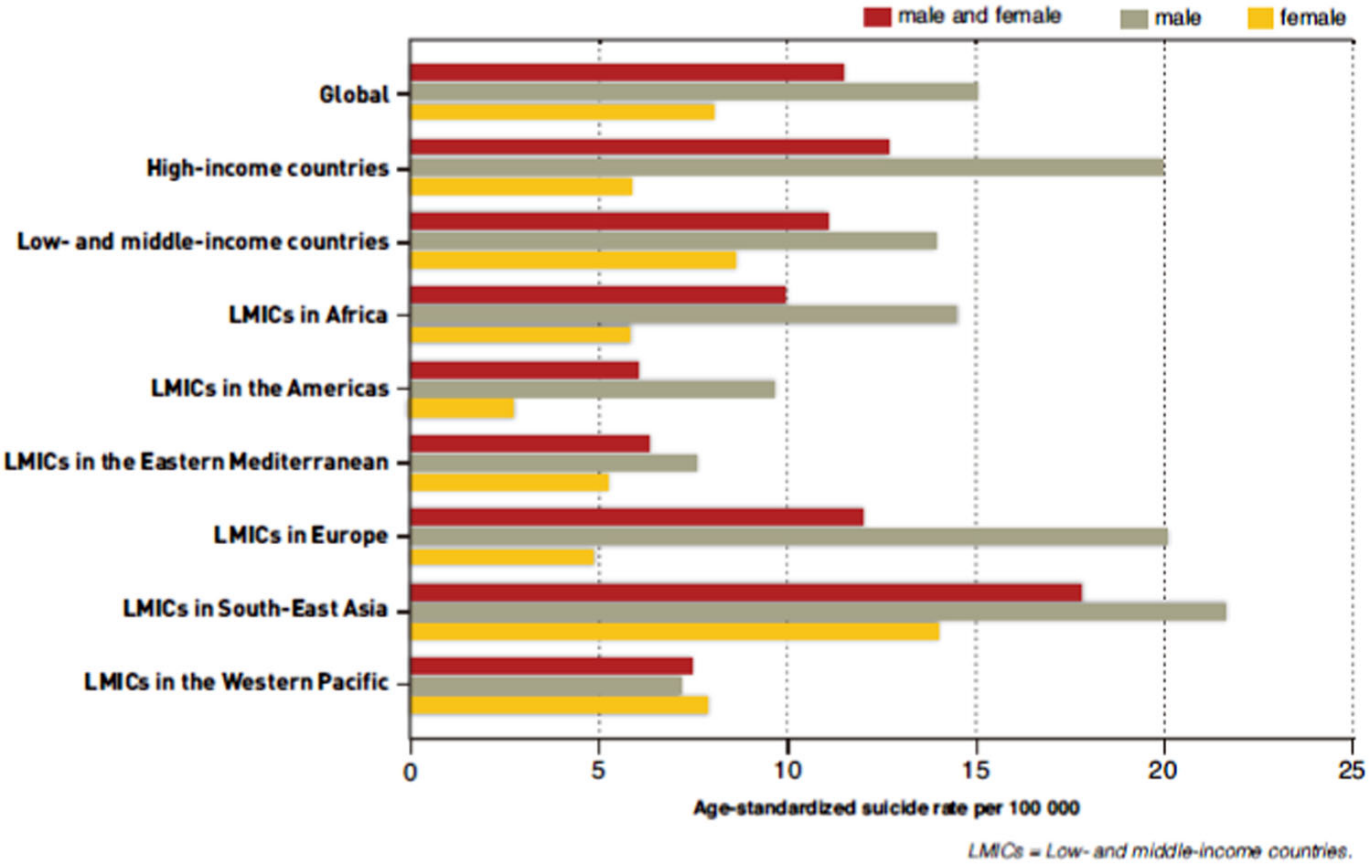
Suicide rates (age-standardised) in different regions of the world, 2012 *Source*: Preventing suicide: a global imperative^1^. Reproduced with kind permission from WHO

After age standardisation, a male residing in an affluent country is more than 3.5 times more likely to die by suicide than a female, a differential that is narrower (1.5 times) in resource-poor states. Within the same gender, regional differentials are revealing: for instance, in 2012, women in the poorer countries of South-East Asia (13.9 suicide deaths/100,000) were more than five times more likely to die from suicide relative to economically comparable regions in the Americas (2.7) (Fig. 2)^1^. Across country comparison within similarly classified regions also reveals striking mortality differentials^1^.

While important in themselves for the quantification of burden, scrutiny of suicide rates by country and time offer important insights into the modifiability and, to an extent, the explicability, of suicide risk, and therefore prevention in principle. Disregarding concerns regarding completeness of suicide death data—likely to be most problematic for suicide relative to other causes of death which do not carry the same degree of social stigma and even illegality in certain countries—these rapid fluctuations in suicide risk are most likely to be ascribed to environmental influences (although gene–environment interactions, yet to be demonstrated, are plausible). Moreover, the downturns in male and female suicide rates during the two world wars, and rises in the 1930s at the time of the Great Depression, coincide with environmental insults or ‘shocks’ that have been replicated in analyses around the global recession of 2008 which itself has been linked to upward inflections in suicide events (see later).

In general, the causes of suicide are poorly understood, particularly when measured alongside chronic diseases such as selected cancers, cardiovascular disease, and diabetes (a diagnosis of which, in themselves, appear to raise suicide risk^4–6^). Knowledge about an array of risk factors for suicide—social, psychological, behavioural, and physiological—should, in principle, enhance our ability to intervene in preventing its occurrence. There is growing evidence that behavioural factors may be related to suicide risk, including poor sleep quality^7^, ease of accessibility to method (e.g., high lethality pesticides particularly in resource-poor countries)^8^, elevated alcohol intake^9^, and media portrayal of suicide (detrimental and protective impacts dependent on depiction^10^). Selected physiological risk indices also seem to be associated with completed suicide, such as raised levels of systemic inflammation^11^ and body weight^12,13^ and, perhaps given its correlation with serotonin, lower blood cholesterol^14^. The purpose of this overview, a version of which features as a book chapter^15^, is to describe the role, if any, of psychological and social characteristics and, in doing so, we focus on those most extensively examined to date in relation to suicide risk (alphabetically ordered): cognitive function; early-life characteristics; personality type; psychosocial stress; serious mental illness, including chronic psychological distress; social integration; and socioeconomic status. We carried out a scoping review to locate existing studies and, where such findings were scarce or require replication owing to a thin evidence base, we conduct new analyses based on data from various cohort studies: the Health Survey for England^16^, the Scottish Health Survey^17^, the Whitehall I^18^, UK Biobank^19^, and the Swedish Conscripts Study^20^. For a summary of the methodologies of these studies, including key instruments, see Box 1. Finally, we also briefly describe progress in identifying psychosocially orientated interventions for suicide prevention.

## Challenges in studying suicide aetiology

The study designs utilised in the examination of suicide aetiology are as wide-ranging as the various types of endpoints (see later)—though perhaps no more so than some other fields of research—including ecological, and, at the level of the individual, cross-sectional, case control, or psychological autopsy studies. That suicide research also spans an array of scientific disciplines, with investigators taking disparate and sometimes unconventional or opaque approaches to study description and data analyses, rather complicates data interpretation.

With death from suicide (completed suicide) being rare, it is commonplace for study investigators to utilise proxies such as hospital admissions after a suicide attempts^21^, self-reported suicide attempt^22^, informant report^23^—most obviously in the context of psychological autopsy—and suicidal ideation^24–26^. Psychological autopsy involves collecting all available information on the deceased using a range of approaches: structured interviews with family members, relatives, friends, or attending healthcare personnel; collating information from healthcare and psychiatric records, and other documents; and forensic examination. While ideation seems to be used as an intermediate indicator of suicide, prospective evidence linking it to subsequent verified suicide events is modest^27^, and the validity of self-reported and proxy-reported measures of suicidal thoughts and behaviour is unclear^28^ with some suggestion of misreporting^29^. Serious mental illness is perhaps the most powerful risk factor for suicide^30^ and, as such, suicide events accumulate more rapidly in people with such disorders than in samples drawn from the general population. As such, for the majority of studies, investigators have used these proxies of suicide outcome in cohorts of people with a diagnosed psychopathology, although less than one-third of people dying by suicide have been under the care of psychiatric services in the previous year with the corresponding figure for lifetime contact being around half^31^.

In this overview therefore, wherever possible, we focus on prospective cohort studies of general population samples—a study design that typically provides stronger evidence of causality than other observational approaches in the context of suicide research—using verified suicide death (completed suicide) or, less commonly, the endpoints of hospital admission/discharge following a suicide attempt. In certain circumstances, completed suicide may approximate to suicide attempts in countries where highly lethal methods are commonly used, such as China and other regions in East Asian where there is a relative ease of access to pesticides. This is analogous to using cancer deaths data to examine aetiology when the malignancy in question has high short-term case fatality (e.g., liver, lung)^32^. As a caveat to our occasional use of suicide attempts data (sometimes referred to as self-harm, although this broader category includes acts carried out without suicidal intent), it is also the case that the epidemiology of completed suicide differs markedly from that of nonfatal suicidal behaviour, most notably in the distribution by age, gender, and methods utilised^33^. Death certification for suicide has a high level of agreement with other sources of evidence (forensic reports, police reports, toxicological and histological data)^34^.

## Psychosocial factors and suicide risk

### Cognitive function

More than 60 years ago, Rook^35^, originally concerned with his hypothesis that physical exertion may be cardio-detrimental, explored causes of death extracted from records routinely kept on Cambridge University alumni. He retrospectively defined sportsmen, his exposed group, as male students with the apparent sporting distinction of representing their university against the traditional rivals of Oxford University; intellectuals were denoted by a distinguished performance in their final undergraduate degree examination; and a random control group was rather unfortunately characterised as ‘… men who have been at the university under survey and had not distinguished themselves sufficiently either academically or as sportsmen …’. On finding that intellectuals were apparently more prone to suicide than other group, in subsequently adopting a more refined study design, Rook noted higher rates of suicide mortality in alumni from various higher education institutions in the UK and the US relative to people of comparable age taken from the census of the general population of England and Wales^36^. More recently, investigators on ecological studies have reported positive correlations between average country-specific scores on standard written intelligence tests and suicide rates^37,38^. While ecological studies may have an important function in the study of suicide—along with natural experiments they may be the only means to examine the impact of population-level exposures—they are nonetheless subject to the concern that group-level results cannot be necessarily extrapolated to the individual—referred to as the ecological fallacy.

**Fig. 3:**
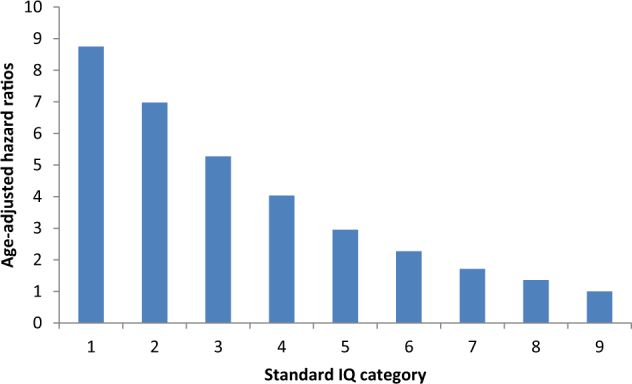
IQ and suicide risk: 17,736 hospitalisations for suicide in 1,109,453 conscripted men *Source*: Based on further analyses of data published elsewhere^20^. IQ category 9 (lowest performance) is the referent

Perhaps the most useful contribution to this area has been individual-level cohort studies from Sweden and Denmark which are based on routinely collected cognition data from school or military records. Participants are then linked to cause of death registries and hospital records using the personal identification number. With follow-up spanning late adolescence through to middle age, one of the lifetime peak periods for the occurrence of suicide is captured and, by the standards of most sample sizes in suicide epidemiology, studies are unusually well powered. Taken together, results from these studies indicate that cognitive function is negatively associated with suicide risk, whereby higher performing school pupils^39^ and young adults who completed IQ tests prior to entry to the military^20,40^ experience a markedly lower risk of completed and attempted suicide up to three decades later. In Fig. 3 we show the results of new analyses of data from an earlier publication^20^, which demonstrate a stepwise relation for suicide hospitalisations across the full IQ range. Of note is the absence of any threshold effect, for instance around average intelligence. The effects estimate in the lowest IQ group, which approaches 10, is of a magnitude rarely seen in modern epidemiology. The strength of this relation implies causality, although in a smaller-scale study, control for childhood educational performance, potentially an indicator of life opportunities, resulted in a null cognition–suicide correlation^39^.

Plausible explanations for these IQ–suicide relationships have been outlined^40^. Low IQ in early life consistently correlates with poor social circumstances^41^ and less favourable health behaviours^42–44^ many decades later. Adjustment for these potential covariates, where available, tends to lead to only modest degrees of attenuation in the effect sizes. Adversity in childhood, other than purely socioeconomic, may also affect IQ or academic performance and future suicide risk, however. For instance, children exposed to violence, either directly as victims or indirectly as witnesses, typically display lower levels of cognitive function^45^. Exposure to violence in childhood has also been reported to increase the risk of suicide ideation or attempts later in life^46^. Although currently speculative, these relations may contribute to associations between IQ and attempted suicide. Perhaps most convincingly, people with lower IQ scores may have lesser problem solving abilities and, in times of crisis, be less well equipped to identify practical solutions to their perceived circumstances^40^. That analyses of the four IQ subtests available in the Swedish conscripts cohort reveal that associations with the logical (problem solving) subscale were strongest offers some support for this suggestion^20^. Findings from studies of children also indicate that higher cognitive ability is associated with a greater internal locus of control^47^, which may lead to lower occurrence of suicidal behaviour^48^.

### Early-life characteristics

At first impression perhaps regarded as a rather eccentric characteristic to study, physical stature peaks around 18 years of age and has utility as a ‘record’ of, among other early-life insults, chronic illness, socioeconomic disadvantage, sub-optimal nutrition, and psychosocial stress^49^. Used for many years in the fields of economic history, anthropology, and anthropometry, its value in epidemiology has arisen from the paucity of longitudinal studies which hold data on prospective collection of these pre-adult factors alongside chronic disease outcomes in older people^49,50^.

**Fig. 4:**
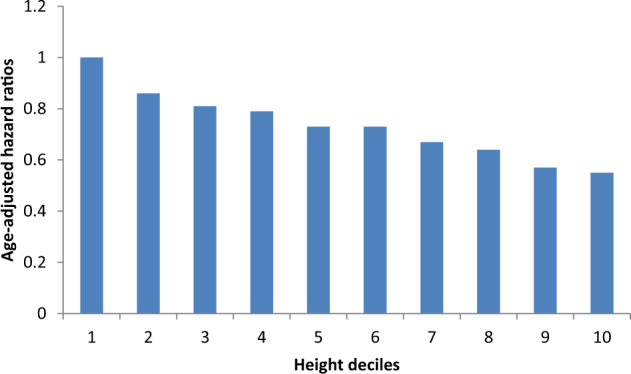
Physical stature and suicide risk: 19,248 suicide hospitalisations in 1,182,114 conscripted men *Source*: Based on further analyses of data published elsewhere^52^. Height decile 1 (shortest) is the referent

A 10-year follow-up of participants in the first Whitehall study of male, London-based non-industrial government workers was among the earlier height–mortality studies^51^. In this early period of mortality surveillance there was an insufficient number of suicide deaths for meaningful analysis, but we have revisited this issue some decades on. We present our results from this cohort of 17,955 men in whom there were 81 deaths ascribed to intentional or undetermined suicide in Table 1 where a graded relationship with height is shown. In new analyses of a group of Swedish conscripts in which the outcome of interest was originally suicide mortality, using hospitalisation following suicide^52^ there was a clear stepwise effect, whereby the shorter study members experienced the greatest risk (Fig. 4). That taller people experiencing lower suicide rates accords with the findings of most^53–57^, if not all^58^, studies.

**Table 1:** Association of psychosocial factors with suicide deaths: 81 suicide deaths in 17,955 men in the original Whitehall study

	Number of suicide deaths	Number of people at risk	Age-adjusted hazard ratio (95% confidence interval)	Mutually-adjusted hazard ratio (95% confidence interval)
*Marital status*				
Married	63	15,806	1.0(ref)	1.0
Single/divorced/widowed	18	2149	2.29(1.36, 3.86)	2.15(1.25, 3.70)
*Physical stature*				
Shortest third(<68 ins)	28	5183	1.0(ref)	1.0
Middle third (68–70 ins)	29	6766	0.76(0.45, 1.27)	0.79(0.47, 1.34)
Tallest third (>70 ins)	24	6006	0.69(0.40, 1.20)	0.74(0.43, 1.30)
*Employment grade*				
High (Admin/Prof/Exec)	57	13,201	1.0(ref)	1.0
Low (Clerical/other grades)	24	4754	1.41(0.85, 2.32)	1.16(0.69, 1.96)

With shorter stature per se very unlikely in itself to be a risk factor for suicide, it is plausible that the characteristics that it proxies are the likely risk indices, including birth characteristics (weight, post-natal growth, birth order), nutrition, illness, psychosocial stress, bullying, and social disadvantage, among others^49^. Aside from its links with height, however, there are several other reasons to implicate early-life characteristics in the development of suicide. First, with suicide, unlike most physical chronic diseases, having a rise in incidence in early adulthood—most notably in men—it is inevitable that some of the causal processes leading to such events occur in childhood. This is not, however, to diminish the importance of life events that may precipitate suicide in later adulthood, such as relationship breakdown, job loss, and financial debt. Second, the occurrence of suicide in adult life does not appear to be fully explained by contemporaneously measured risk factors^59^. Third, some of the known or potential adult risk indices for suicide—poverty^60^, psychological distress^61^, personality type^62^, low cognition^63^—tend to ‘track’ across the life course such that children with unfavourable levels of these characteristics are more likely to become adults with unfavourable levels.

In this context, considering social circumstances or adversity more broadly, investigators have prospectively examined the predictive capacity of parental socioeconomic disadvantage, lower performance on cognitive testing, psychological distress, death of one or both parents from suicide and other causes, parental separation, periods spent in public care, and bullying^64,65^. Birth cohort studies, provided samples are sufficiently large, have the potential to explore these relationships, and the 1958 birth cohort study (NCDS)^66^ is unusually well placed in this regard. In that study, 50 years of mortality surveillance revealed relations of suicide with a series of psychosocial factors, including higher birth order (potentially a proxy for attention lavished, bullying by older siblings, maternal depression which is more common in second and third pregnancies), poor daytime bladder control after 3 years of age, internalising and externalising behaviours, a father in a manual occupational social class, parental loss, neglected appearance, and contact with social services that might extend to institutionalised care. Selected analyses were, however, underpowered, as evidence by the wide confidence intervals, and the absence of an IQ–suicide link in this study does not accord with findings from larger cohort studies^20,40^.

The predictive capacity of childhood bullying for later life suicide is becoming increasingly well explored. While being the victim of bullying, as opposed to being the perpetrator, seems to be related to a higher risk of subsequent self-reported suicide, planning and ideation^67^, as discussed, the validity of these endpoints is uncertain, and recall of pre-adult victimisation in cross-sectional studies of older adults raises further concern regarding the accuracy of this information. In the few prospective birth cohort studies with verified suicide outcomes, being a victim of bullying appears to confer an elevated risk in a sample drawn from the UK^64^, while in a Finnish study this effect was lost after multiple adjustment for related risk indices for men who, as boys, had been bullied, but held for women^68^.

### Personality type

As with many of the potential suicide risk factors featured in this review, personality disposition has most commonly been related to total mortality, cardiovascular disease, cancer^69^, and, more recently, mental health^70^. Impulsivity is perhaps the personality disposition most frequently theorised as being linked to suicide risk^71^. While early studies of personality type used unvalidated assessment tools, results are nonetheless intriguing and bear description. In a 13-year follow-up of 50,465 Swedish men who had been conscripted into military service (women were not required to participate), on the basis of questionnaires response and an interview with a trained psychologist, those with poor emotional control, which is akin to high impulsivity, and lower social immaturity at around 18 years of age experienced an increased risk of subsequent completed suicide^72^. In extended follow-up of Harvard University alumni, those men who reported frequent periods of feeling particularly self-conscious were, based on our own computation of the results presented in that paper, seemingly four times more likely to experience death from suicide than those who had no such feelings^73^. For context, similar results were apparent when self-report of physician-diagnosed depression was the outcome of interest.

In Table 2, we describe four cohort studies, identified through our scoping review, in which investigators have utilised standard, validated personality questionnaires—the Eysenck or Minnesota Multiphasic Personality Inventory—to quantify associations of suicide or suicide attempts with one or more of what are now regarded as the major personality traits: conscientiousness, agreeableness, neuroticism, openness, and extraversion^74^. No single study explored the impact of all five personality dispositions: two assessed extraversion/introversion^75,76^ and three measured neuroticism^22,75,77^. However, in two of these studies^22,77^, the suicide outcome was based on self-report—included here for the purposes of completeness—which raises the usual concerns regarding validity. In the only study with verified suicide outcomes, a score of ≥9 on the neuroticism subscale from the Eysenck questionnaire was associated with a more than doubling of the risk of suicide mortality in a Japanese general population (hazard ratio; 95% confidence interval: 2.39; 1.37; 4.18), even after statistical control for a range of covariates^75^. Using the same thresholds to denote extraversion, results were less convincing and statistical significance at conventional levels was not apparent (1.37; 0.74; 2.56). In that study, despite the novelty of these results on the full cohort, the primary aim of the authors was to ascertain if the personality–suicide relation was modified by the financial crisis in Japan. Partitioning follow-up at 1998 when this environmental shock began in Japan, the authors speculated that neurotic individuals would be most prone to its impact in the following 10 years of mortality surveillance relative to the preceding eight. This was supported by their analyses showing that, compared with the lowest category, the hazard ratios for the highest neuroticism group increased from 0.66 (0.13; 3.37) to 2.45 (1.26; 4.74) during this period^75^.

**Table 2:** Personality and suicide risk: summary of findings from general population-based cohort studies

Study name^ref.^	Study design and sample	Assessment of personality type and suicide	Results
Australian Twin Registry^77^	Prospective cohort study of twins initially surveyed in 1979/1991 and again in 1989/1994	Eysenck Personality Questionnaire. Self-reported serious suicide attempt	Odds ratio (95% confidence interval) for 75th vs. 25th centile for neuroticism: 1.99 (1.42; 2.79)
No study name^76^	Prospective cohort study of US state university alumni. Four analytical groups: suicide completion group (*N* = 44), clinically depressed group (*N* = 39), control deceased group (*N* = 39), control living group (*N* = 39). Age at baseline and duration of follow-up not reported	Minnesota Multiphasic Personality Inventory. Cause of death from death certificate	Social introversion scores were higher in suicide completion group vs. deceased control group (*p* = 0.0015)
Christchurch Health and Development Study^22^	Prospective cohort study within a single New Zealand city, general population; *N* = 881 to 1025 (dependent on analyses)	Eysenck questionnaire at age 14 years. Self-reported suicide attempt between 14 and 21 years of age	Neuroticism was positively related to suicide attempt (betta coefficient 0.059, *p* < 0.05)
Miyagi Cohort Study^75^	Prospective cohort study sampling participants from the Miyagi region (northern Japan); general population; *N *= 29,432 aged 40–64 years at baseline followed for a maximum of 18 year	Eysenck Personality Questionnaire (subscale range: 1–12 with higher number indicating greater degree of a given personality type). Cause of death from death certificate	Age-adjusted and sex-adjusted hazard ratio (95% confidence interval) for ≥9 vs. ≤3: 1.37 (0.74; 2.56) for extraversion, and 2.39 (1.37; 4.18) for neuroticism. Little impact after further statistical adjustment

Given the dearth of studies that have been used to investigate the association of different personality types with completed suicide, we carried out new analyses using the UK Biobank study. We found that neuroticism measured using the 12-item Eysenck Personality Questionnaire-Revised Short Form—the only personality disposition captured in the study—was positively related to suicide risk (age-adjusted and sex-adjusted hazard ratio per 1 standard deviation increase; 95% confidence interval: 1.72; 1.36; 2.19) (Table 3). The mechanisms that may explain the link between neuroticism and completed suicide are currently unclear. One possibility is people displaying higher levels of neuroticism are more likely to be vulnerable to the depression-inducing effect of stressful life events than those with lower levels^78^. Expressed differently, and as outlined above, lower levels of neuroticism may buffer the impact of adverse psychological experiences. More research in this field would add clarity. In particular, the extent to which these personality traits may simply be a reflection of poor mental health is moot.

**Table 3:** Association of psychosocial factors with suicide mortality: up to 149 suicide deaths in 449,073 participants in UK Biobank

		Number of suicide deaths	Number of people at risk	Age- and sex-adjusted hazard ratio (95% confidence interval)
Psychological distress	1 (low)	48	177,893	1.0 (ref)
(PHQ-4)	2	45	163,630	1.04 (0.69, 1.56)
	3	56	107,550	1.90 (1.29, 2.81)
P for trend				0.002
Per 1-SD (2.11 points) increase		149	449,073	1.35 (1.20, 1.52)
Psychiatric consultation	No	105	441,285	1.0 (ref)
	Yes	66	57,681	5.01 (3.68, 6.82)
Neuroticism	1 (low)	21	107,993	1.0 (ref)
	2	35	128,738	1.57 (0.95, 2.69)
	3	75	164,694	2.74 (1.68, 4.46)
P for trend				<0.0001
Per 1-SD (3.27 points) increase		131	321,456	1.68 (1.43, 1.96)

### Psychosocial stress

Psychosocial stress, which may be a precipitant of psychological distress, has been most well explored in the context of chronic disease endpoints, particularly cardiovascular disease^79^. We were able to identify four prospective cohort studies with suicide as an outcome (Table 4). The two studies offering the greatest number of suicide cases utilised unvalidated questionnaire assessment of stress at home only^80^ or both home and work^81^. Each found a ‘U’-shaped relationship whereby both higher and lower levels of stress was associated with the greatest risk of study members taking their own lives, while moderate levels were linked to the lowest rates. In the two other studies, investigators used either the Karasek questionnaire^82^ or a derivation of it^83^ to quantify job-related psychosocial stress. In doing so, in a Japanese population, there was a suggestion that low job control was associated with a 4-fold elevation in suicide risk, with no apparent effect for job demand^83^, while in German workers, after deriving a job strain index by dividing job demand by job control—higher scores are regarded as being disadvantageous—there was no indication of a relation with completed suicide^82^.

**Table 4:** Psychosocial stress and suicide risk: summary of findings from general population-based cohort studies

Study name^ref.^	Study design and sample	Assessment of psychosocial stress and suicide	Results
Nurses’ Health Study^81^	Prospective cohort study of 94,110 US married, female registered nurses aged 30–55 years at baseline followed for maximum of 14 years giving rise to 73 suicide deaths	Experience of stress at home and work, categorised as ‘minimal’, ‘light’, ‘moderate’, or ‘severe’	‘U’-shaped relation: multiply-adjusted hazard ratio (95% confidence interval) for suicide risk for women reporting minimal (2.1; 1.0–4.5) or severe stress (3.7; 1.7–8.3) in the home, and minimal (2.4; 0.9–6.1) or severe stress (1.9; 0.8–4.7) in the workplace
Fukuoka region study^80^	Prospective cohort study of 13,259 people (7337 women) from the general population aged 30–79 years at baseline followed for a mean of 7.4 years giving rise to 48 suicides deaths	Stress was assessed using a non-standard questionnaire concerning ‘home life’ in the prior year (four categories of frequency). Death from suicide from cause of death registers	Multiply-adjusted hazard ratio (95% confidence interval) for suicide risk for people reporting occasional stress (2.9; 1.2; 6.9) and no stress (3.1; 0.8; 11.8) relative to very occasional group
Jichi Medical School Cohort Study^83^	Prospective cohort study of 3125 men aged ≥65 years followed for a max 10 years giving rise to 14 suicide deaths	Job control and job demand assessed using the WHO MONICA Psychosocial Study Questionnaire. Death from suicide from cause of death registers	Multiply-adjusted hazard ratio (95% confidence interval) for suicide risk for low job control relative to high: 4.10 (1.31; 12.83), and for high job demand relative to low: 0.73 (0.22; 2.38)
MONICA Augsburg project^82^	Prospective cohort study of 6817 men and women aged 25–74 years followed for a mean of 12.6 years giving rise to 28 suicide deaths (2 in women)	Job strain as assessed by the Job Content Questionnaire (Karasek). Death from suicide obtained by data linkage	Multiply-adjusted hazard ratio (95% confidence interval) for suicide risk in the higher job strain group relative to low/intermediate: 1.67 (0.76; 3.68)

### Serious mental illness and chronic psychological distress

Using people institutionalised in New York City care facilities for treatment of serious mental disorders as an ‘exposed’ group, by comparison with rates in the general populations, Malzberg, a psychiatrist, in a series of articles over 80 years ago, was able to examine the relation of serious mental disorders and mortality risk^84,85^. He estimated that these patients had a life expectancy, on average, 14–18 years shorter than their counterparts in the general population who are largely free of such conditions. Current estimates suggest this differential may be increasing^86,87^.

Data from numerous cohort studies—often constructed using data linkage—have been used to examine the relation of serious mental disorders, collectively and individually, with suicide mortality. Unsurprisingly, the magnitude of the effect estimates vary markedly by setting (primary care cohorts, hospitalised patients) and the diagnosis of mental disorder. Thus, hazard ratios for mental health problems in people being treated in the community via their general practitioners^6^ are lower than for institutionalised patients^30^. While around a doubling in risk of taking one’s own life is evident in people with post-traumatic stress disorder, individuals with borderline personality disorder experience a more than 10-fold increase^30,88^ with some meta-analyses suggesting this figure may be as high as an unlikely 50 times^89^. Even the more moderate estimates are large effects by modern day standards and approximate, for instance, to those described for smoking and lung cancer^90^.

While such effect estimates suggest that the relation between serious mental disorder and suicide may be causal—control for unmeasured or unknown confounding variables is unlikely to explain the high magnitude of such relationships—with serious mental disorders being comparatively rare, population impact, the product of the prevalence of the exposure and the hazard ratio, is likely to be lower than, for instance, physical inactivity and heart disease, or smoking and lung cancer. In new analyses, we therefore examined whether suicide risk was also elevated for commonly occurring but less severe mental health problems as denoted by chronic psychological distress. Also termed common mental disorder, psychological distress is a combination of depression and anxiety and should not, as is often the case, be confused with psychosocial stress (described earlier) as has been the case^91,92^. In UK Biobank, study members self-declared whether they had ever been under the care of a psychiatrist—in the UK National Health Service this would ordinarily have followed referral by a general practitioner in a country in which private healthcare is rare, as opposed to self-referral. Participants also completed the four item Patient Health Questionnaire (PHQ-4) scale of psychological distress, scores from which were categorised into three groups, low (score: 0), moderate (1–2), high (≥3), with a higher value denoting greater severity. As shown in Table 3, in around 500,000 people in this study in whom 6 years of follow-up gave rise to 98 suicide deaths, consistent with existing evidence, serious mental disorder at some point in the life course was associated with a 6-fold increased risk of suicide (5.82; 3.90; 8.70). There was also around a doubling of suicide risk in study members reporting symptoms of moderate-to-severe distress, though intermediate levels were unrelated. In better powered studies, including an individual participant data meta-analysis of data from the Health Surveys of England and Scottish Health Surveys, graded effects across the full distress continuum have been seen^93^.

Multiple mechanisms have been advanced to explain the link between psychological distress and suicide risk, including direct (biological), indirect (behavioural) pathways plus their interaction, and a discussion of them would perhaps warrant a separate review in its own right. This notwithstanding, distress may lead to alcohol abuse and depression; and alcohol use itself may disinhibit, raise levels of impulsivity and/or impair the cognitive processes that may lead to the initiation of a suicide attempt. Speculation about mechanisms aside, these findings raise the question whether healthcare professionals should pay attention to suicide risk at distress levels lower than current recommendations suggest.

### Social integration

**Fig. 5:**
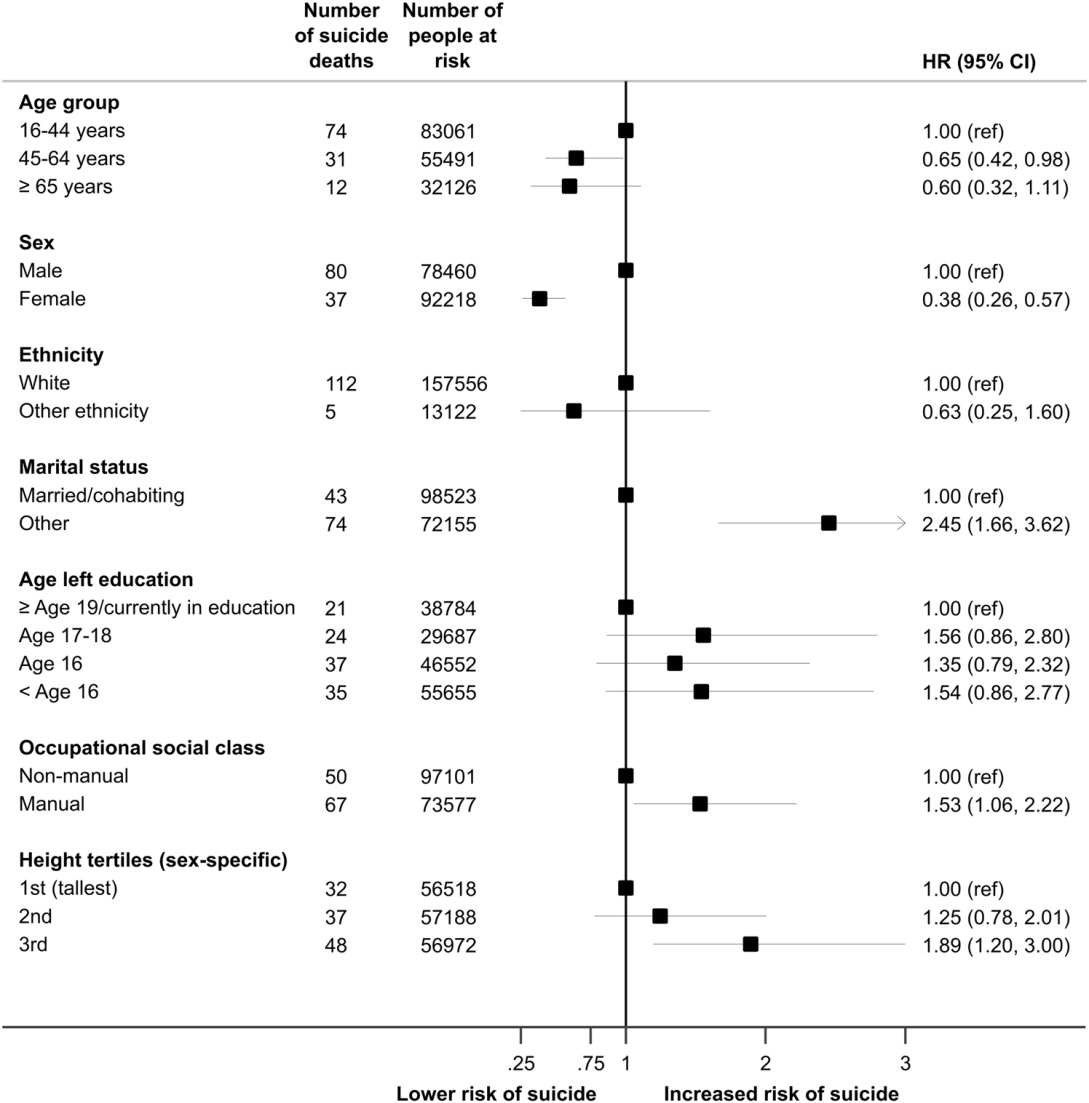
Association of psychosocial factors with suicide deaths: 117 suicide deaths in 170,678 individuals from the Health Survey for England and the Scottish Health Survey *Source*: Based on further analyses of data published elsewhere^93^

While the first empirical examination of a relationship between a low number of friends and suicide risk was probably undertaken three decades ago^72^—using a subgroup of participants from the Swedish Conscripts study (Box 1)—the majority of the work in this area has utilised marital status as a proxy for social support. The consistent observation is that people who were married or cohabited at study baseline in cohort studies are less likely to subsequently take their own lives relative to people who live alone or the unmarried^14,94^. In those studies well powered enough to stratify by gender, effects appeared to be confined to men^95,96^. The protective effect of cohabitation or being married accords with new analyses herein based on a pooling of data for men and women from the Health Survey for England and the Scottish Health Survey (Fig. 5), and in the all-male original Whitehall cohort study (Table 1). In these analyses, however, we were insufficiently powered to explore gender-specific effects and also unable to distinguish between the type of union or cohabitation. In a rare example of a study that did, utilising routinely collected data from Scandinavia^97^, with opposite-sex married persons as the referent category, there was a raised risk of total mortality for same-sex married women (hazard ratio; 95% confidence interval: 1.89; 1.60; 2.23), that was largely generated by strong relationships for suicide (6.40; 3.42; 12.00). Similar observations were made in men. Explanations for these results include parental rejection and societal stigmatisation of people in same-sex unions.

Recognising that marital status and cohabitation fail to fully capture social integration, Tsai and others^98,99^ utilised a composite measure based on a 7-item index first developed by Berkman^100^ that, in addition to marital/cohabitation status, also incorporated social network size, frequency of contact, religious participation, and involvement in other social groups. Set within an unusually large bespoke occupational cohort study from the US (as opposed to one generated purely from data linkage) which provided a high number of suicide deaths to facilitate analyses, and based on over two decades of mortality surveillance, the authors found that men who were socially well-integrated had around half the risk of suicide relative to those who were less well connected^98^. Of the individual elements of the derived index for social integration, marital status (again), social network size, and religious service attendance seemed to be generating the association with suicide. The lack of availability of baseline data on mental health—mental illness may impair the building of a support network^101^ or be detrimental to its maintenance, and people with mental health problems, as described, experience an elevated rate of suicide^30^—was addressed by the same authors in a separate study of women where similar patterns of association appeared to hold^99^.

### Socioeconomic status

Socioeconomic circumstances are denoted by an array of characteristics including, at the level of the individual, occupational social class (most common in studies of UK populations), education, income, and race (more common in studies of US populations), and at the area or group level, neighbourhood deprivation^102^. Epidemiologists tend to use the collective terms socioeconomic status or socioeconomic position, while social scientists often refer to social class even when not referring to occupation per se. When using occupation as an indicator of socioeconomic status, interpretation of the relationship with suicide is to an extent complicated by access to lethal means across different professions that are perhaps not socially patterned. For instance, it would seem likely that farmers have ready access to firearms, and physicians to potentially lethal medicines.

The study of the link between socioeconomic status and suicide was probably first initiated by Morselli^103^, followed by Emile Durkheim in the 19th century^104^, Cavan in 1920s Chicago^105^, and latterly Sainsbury in London^106^. In a recent systematic review of studies utilising various research designs, drawing on clinical and non-clinical populations, people with a lower income, a more basic education, or a less prestigious occupation have around a doubling of suicide risk with weaker associations apparent in women^107^. Results appearing subsequently provide support for these associations^14,108^, as do the findings of our own analyses of new data from the Health Surveys for England and the Scottish Health Survey (Fig. 5) where higher occupational social class and education were related to lower suicide rates, although statistical significance at conventional levels was not always apparent. While higher employment grade in the Whitehall I study appeared to show some protection again suicide (Table 1), the magnitude of the relationship was modest.

Although rates of male suicide are somewhat lower in resource-poor countries, owing to greater populations numbers, the absolute occurrence is higher such that countries in this group account for three-quarters of all suicide deaths worldwide^1^. In reviews focusing on studies set in these societies, alternative markers of socioeconomic position are necessarily utilised (e.g., asset ownership, quality of household construction), yet similar patterns of association are seen: lower socioeconomic position is typically associated with elevated suicide risk and these effects seem to hold whether intermediate (suicidal ideation, behaviour) or endpoint (deaths from suicide) indicators are the outcomes of interest^109,110^.

In contrast to other health outcomes such as chronic disease—most obviously cardiovascular disease^111^ and cancer—for suicide, the time between exposure and outcome may be much shorter because suicide is a behaviour rather than a chronic disease process, and some exposures, such as job loss and relationship breakdown, have acute effects on psychological distress. For instance, expressed simplistically, rapid onset of acute financial hardship, or relationship breakdown may precipitate mental health problems which subsequently give rise to suicide. In this context, the impact on suicide risk of the recent worldwide economic crises can be examined by using a time series approach where death rates are compared pre and post-economic downturn. Based on such data, following the global economic shock of 2008, as evidenced by rises in unemployment, debt, and home repossessions, there was profound impact on economies in multiple nations only previously seen during the Great Depressions of the 1920 and 1930s. Relative to expectations had prior mortality trends continued, there was a suggestion of marked elevations in rates of death from suicide^112,113^. Estimates in the UK attribute 1000 suicide deaths to the recession^33,114^, which, based on the multiplier used earlier, would equate to an additional 30–40,000 attempts on life. Stratification of data by age show that peri-recession, young people experienced particularly high levels of job losses and unemployment suggesting a potential for greater suicide risk in this group. Cross-national studies reveal the apparent impact of recession on suicide rates varies by location^112^. While these country-to-country differentials are partially ascribed to a heterogeneity in levels of recession and associated unemployment, they are also likely to be due to variation in the systems in place to buffer the influence of a recession, including composition of existing welfare systems (e.g., more favourable unemployment benefits), investment in active labour market programmes (e.g., job search assistance, apprenticeships, subsidised employment), and, more generally, a rapid response at a policy level which aim to support those affected by recession^115^. Of note, during the same period that rates of people killing themselves increased, there was a decline in road traffic accidents^115^. This was presumably because avoidance of car use owing to running costs, repossession of existing vehicles, or there being fewer heavy goods vehicles on the road.

## Suicide prevention in the context of psychosocial risk factors

Most of the interventions for effective suicide reduction do not focus on psychosocial factors, partly because evidence linking psychosocial factors with suicide risk is, as we have demonstrated herein, in its infancy, or, where it does exist (e.g., cognition, personality type), selected factors are not readily modifiable. Out with the sphere of psychosocial factors, there is reasonable evidence for suicide prevention via method restriction (access to means), including state-level regulation of firearms and pesticides^116^, but also access to ‘hot spots’ such as bridges, buildings, and railway lines;^117^ ‘gatekeeper’ training, whereby lay and professional people receive brief instruction in the warning signs of a suicide and how to react;^118,119^ liaison with the media;^10^ and, based on ecological evidence, state-initiated declines in alcohol consumption^120^.

Of more relevance to the psychosocial factors outlined herein, suicide prevention strategies have attempted to promote awareness in all strata of society, with the aim of reducing factors that increase risk, such as social isolation, mental health problems, and increasing factors that promote resilience or coping, such as individual, family, and community connectedness. Evaluated interventions include: school-based suicide awareness programmes where the quality of most studies does not permit clear conclusions;^121^ cognitive behavioural therapy in reducing recurrence of self-harm, though impacts on suicide are unclear;^122^ antidepressant drug treatment whereby protection against suicide seems to be apparent in older adults, with a reverse gradient apparent in younger people;^123^ measures to prevent bullying;^124^ and social welfare measures to provide better support to populations experiencing financial hardship such as recession and job loss^125,126^.

## Conclusions and future research directions

Examination of the relation of psychosocial risk factors with suicide is hampered by a series of issues. A low number of cases in published studies is commonplace, and the risk factor data collected are also often of modest or little utility in suicide research. Thus, the better powered studies, which are typically derived from data linkage, while having ample suicide events, are insufficiently well characterised for potential risk indices; the reverse is true of birth cohort studies.

The context of the research may have an impact on the association of psychosocial characteristics and suicide risk, at least for those risk factors that are not causally linked with the taking of life. Thus, this relationship may vary by epoch, country, economic development, or cultural setting. The utilisation of data from these different contexts where potential risk factors have different confounding structures^127^ may be particularly helpful.

These points notwithstanding, in the present overview, the strength of the evidence of an association of selected psychosocial factors with suicide was variable. There is good evidence for low socioeconomic status (including job loss and debt), poor cognition (striking effects in Swedish conscripts), and mental illness/psychological distress. The role of personality type, psychosocial stress, and pre-adult factors, aside from height, such as bullying is less clear. Further, while these risk factors may have some utility in offering insights into suicide aetiology, not all are of value to suicide prevention, either because they are markers of other characteristics (e.g., height) or do not readily lend themselves to modification (e.g., cognitive function, personality type)—although the latter may have value in high-risk (targeted) approaches to suicide prevention. Other potential risk factors, such as poverty, social isolation, and mental health are already firmly on the policy agenda either because they are important health entities in their own right, or they have been linked with other health outcome (poverty and life expectancy; social isolation and dementia).

### Box 1 Profile of cohort studies contributing to new analyses herein

The Whitehall I Study: Established as a screening study for trials of treatment for smoking cessation and diabetes^18^, the Whitehall I Study has been most frequently utilised as a cohort study in its own right. Data were collected on 19,019 male, non-industrial, government employees aged from 40 to 69 years when examined 1967–1970 in London (UK), representing a 77% response. This involved the completion of a study questionnaire and participation in a medical examination. In brief, the questionnaire included enquiries regarding civil service employment grade (an indicator of socioeconomic status), marital status, and health behaviours. Physical stature was measured directly. Men were traced using the UK National Health Service Central Registry which also provided data on cause of death, including suicide.

UK Biobank: This is a UK-wide, ongoing, prospective cohort study established to explore gene × environment interactions in the context of chronic disease of major public health importance. Described in detail elsewhere^19^, between 2006 and 2010, 502,649 participants aged 37–73 years attended various geographically disparate research clinics. Participants populated a questionnaire, underwent an interview, and took part in various physical assessments. Study members reported consultation with a psychiatrist, and psychological distress was measured using the 4-item version of the Patient Health Questionnaire (PHQ-4) with total scores ranging from 0 to 12 (higher scores denote greater distress)^128^. Neuroticism was measured with the 12-item Eysenck Personality Questionnaire-Revised Short Form^129^. A notable feature of the study is the extremely low response proposition (<10%) resulting from the decision not to reissue requests to participate to non-responders (this suggests that 5 million people were invited to participate). Study participants were traced using the UK National Health Service Central Registry which also provided data on cause of death, including suicide.

The Health Surveys for England and the Scottish Health Surveys: The Health Survey for England (HSE)^16^ and Scottish Health Survey (SHS)^17^ are a series of geographically representative health examinations of people from the general population. Between 1994 and 2008, 16 independent, cross-sectional, and methodologically near-identical studies were conducted on either an annual (HSE; *N* = 13) or occasional basis (SHS; *N* = 3). The original purpose of these studies was to monitor secular trends in health and related behaviours. A total of 199,504 men and women, aged 16–107 at baseline, were surveyed. Socioeconomic status (including age of leaving education, occupation socialclass^130^) marital status, and ethnicity were obtained via self-report, and height was measured directly. Consenting study members linked to national health registers for vital status, including suicide death^131^.

The Swedish Conscripts Study: The record linkage procedures used to generate this cohort have been reported previously^20^. In brief, the cohort comprised all non-adopted men born in Sweden from 1950 to 1976 with both biological parents identified in the multi-generation register. Unique personal identification numbers were used to link the people in this register with the population and housing censuses records (1960 and 1970), and military service conscription, cause of death, and national hospital discharge registers, resulting in 1,379,531 successful matches. The military service conscription examination involves a structured, standard medical assessment of physical (including height), mental health, and cognitive function. IQ was measured by four written subtests representing verbal, logical, spatial, and technical abilities^40^. Suicide information was extracted from hospital admissions data (1969–2006).

In all studies, the relevant ethical approvals were obtained and participants provided written informed consent with the exception of the original Whitehall study where, given the era of baseline data collection, it was not required. Hazard ratios were exclusively computed using the Cox regression technique.
